# Precise location of three novel linear epitopes using the generated monoclonal antibodies against the Knob domain of FAdV-4 surface structural protein, fiber1

**DOI:** 10.3389/fcimb.2024.1468428

**Published:** 2024-09-18

**Authors:** Yongxiao Chai, Qianyue Jin, Rongfang Zhu, Zhenhua Guo, Qingxia Lu, Shujun Chai, Yunrui Xing, Lu Han, Guangxu Xing, Gaiping Zhang

**Affiliations:** ^1^ College of Veterinary Medicine, Northwest A&F University, Yangling, China; ^2^ Key Laboratory of Animal Immunology, Institute for Animal Health, Henan Academy of Agricultural Sciences, Zhengzhou, China; ^3^ Henan Husbandry Technology Promotion Station, Zhengzhou, China; ^4^ Jiangsu Co-Innovation Center for the Prevention and Control of Important Animal Infectious Diseases and Zoonoses, Yangzhou University, Yangzhou, China

**Keywords:** FAdV-4, fiber-1 knob protein, monoclonal antibodies, B cell epitopes, structural analysis

## Abstract

**Background:**

Fowl adenovirus serotype 4 (FAdV-4) is the main pathogen of hepatitis-hydropericardium syndrome (HHS), which brings huge economic losses to the poultry industry worldwide. Fiber-1 protein plays an important role in viral infection and pathogenesis by binding directly to cellular receptors of FAdV-4. In particular, the knob domain of fiber-1 protein has been reported to induce the production of neutralizing antibodies and arouse protection against the lethal challenge of chickens with FAdV-4.

**Methods:**

The fiber-1 knob (F1K) protein was expressed in a prokaryotic expression system and purified using Ni-NTA affinity chromatography. Monoclonal antibodies (mAbs) against FAdV-4 were generated by immunizing BALB/c mice with the purified F1K protein and screened using a series of immunoassays. Potential B cell epitopes on the knob domain of fiber-1 protein were mapped using enzyme-linked immunosorbent assay (ELISA) and dot-blot. Precious location and crucial amino acids of the identified epitopes were determined using peptide array scanning, truncations and alanine-scanning mutagenesis. The epitopes were analyzed and visualized on the knob trimer of FAdV-4 fiber-1 protein using the PyMOL software.

**Results:**

Water-soluble recombinant fiber-1 knob (F1K) protein was obtained with the assistance of chaperone. Four monoclonal antibodies (5C10, 6F8, 8D8, and 8E8) against FAdV-4 were generated and characterized using indirect ELISA, Western blot, dot-blot, and immunological fluorescence assay (IFA). The mAbs were demonstrated to be from different hybridoma cell lines based on the sequences of the variable regions. Meanwhile, three distinct novel linear B-cell epitopes (^319^SDVGYLGLPPH^329^, ^328^PHTRDNWYV^336^, and ^407^VTTGPIPFSYQ^417^) on the knob domain of fiber-1 protein were identified and the key amino acid residues in the epitopes were determined. Structural analysis showed that the two adjacent epitopes ^319^SDVGYLGLPPH^329^ and ^328^PHTRDNWYV^336^ were exposed on the surface of the fiber-1 knob trimer, whereas the epitope ^407^VTTGPIPFSYQ^417^ was located inside of the spatial structure.

**Conclusion:**

This was the first identification of B-cell epitopes on the knob domain of fiber-1 protein and these findings provided a sound basis for the development of subunit vaccines, therapeutics, and diagnostic methods to control FAdV infections.

## 1 Introduction

Hepatitis-hydropericardium syndrome (HHS), which is mainly caused by fowl adenovirus serotype 4 (FAdV-4), has resulted in significant economic losses in the poultry industry worldwide (Mazaheri et al., 1998; Schachner et al., 2018). It was first reported in Pakistan in 1987 and spread rapidly to other poultry-rearing countries including Mexico, the USA, Russia, Poland, Chile, Korea, India, and Japan (Anjum et al., 1989; Asrani et al., 1997; Dahiya et al., 2002; Mase et al., 2009; Choi et al., 2012; Niczyporuk, 2016). Since 2015, outbreaks of HHS have been reported in many regions of China (Liu et al., 2016; Zhang et al., 2016; Niu et al., 2022). The causative pathogen was identified to be a novel genotype of FAdV-4, which possessed high pathogenicity and led to a mortality of 20–80% in 3 to 6-week-old broiler chickens (El-Shall et al., 2022).

Fowl adenoviruses (FAdVs) belong to the family Adenoviridae, genus Aviadenovirus, and are generally grouped into 5 species (FAdV-A to FAdV-E) with 12 serotypes (FAdV-1 to 8a and 8b to 11) based on serum cross-neutralization tests (Hess, 2000; Balamurugan and Kataria, 2004). The viral capsid of FAdVs consists of three major structural proteins: hexon, penton base, and fiber (Wang et al., 2023). FAdV-4 is a member of FAdV-C species which comprises two fiber proteins: fiber-1 and fiber-2 (Marek et al., 2012). The two fiber proteins are exposed on the surface of the capsid structure of FAdV-4 and both contain a tail, shaft, and knob domain (Sohaimi and Hair-Bejo, 2021). Previous studies have reported that fiber-1 protein was a key factor for directly mediating FAdV-4 adsorption onto host cells during the infection (Wang and Zhao, 2019; Zou et al., 2021; Lu et al., 2022). It has been demonstrated that fiber-1 could interact with the chicken coxsackie and adenovirus receptor (CAR) to promote viral entry and establish infection (Pan et al., 2020). And the receptor-binding site of fiber-1 protein has been located on the knob domain which locates outside of fiber-1 protein and exists as a trimer on viral particles (El Bakkouri et al., 2008; Baker et al., 2019). Although a recent study has shown that coexpression of the shaft and knob domains in LMH cells was necessary for resisting FAdV-4 infection, it also found that the sera against the knob domain could effectively neutralize the virus and block the infection, providing efficient protection against the lethal challenge of chickens by FAdV-4 (Wang et al., 2020b). Taken together, these studies confirmed the significant roles of fiber-1 protein and its knob domain in mediating the infection of FAdV-4 and suggested a promising application of knob domain-based subunit vaccine against FAdV-4.

Epitopes are the functional units on the antigens that can be specifically recognized by B-cell receptors and antibodies (Sanchez-Trincado et al., 2017). They are the essential motifs to efficiently stimulate the production of adaptive immune responses (Palatnik-de-Sousa et al., 2018). The identification of B-cell epitopes is important for developing diagnostic assays and designing epitope-based vaccines as well as therapeutics to prevent the spread of diseases (Wang et al., 2020a; Liu et al., 2021b). It has been reported that the peptide stretch of 1-225 aa in penton base protein was the most promiscuous B-cell and T-cell epitope region and equal protection levels were reached after immunization of chickens with the peptide stretch, full-length penton base protein, and the commercial inactivated vaccine against FAdV-4 (Aziz et al., 2019). Another study designed a chimera containing epitopes of fiber proteins from two distinct serotypes, FAdV-4 and FAdV-11, and found that vaccination with the chimera simultaneously protected chickens against HHS caused by FAdV-4 and inclusion body hepatitis caused by FAdV-11 (De Luca et al., 2022). To date, the distribution of B-cell epitopes on fiber-1 protein of FAdV-4, especially the knob domain, has not been studied.

In this study, recombinant fiber-1 knob (F1K) protein was co-expressed with chaperones in Escherichia coli (*E. coli*) cells. It was found that the expression of chaperone significantly increased the production of water-soluble F1K protein. After purification using Ni-NTA chromatography, F1K protein was used to immunize BALB/c mice. Four monoclonal antibodies (mAbs) against FAdV-4 were generated using hybridoma technology. Western blot and immunological fluorescence assay (IFA) showed that the mAbs (5C10, 6F8, 8D8, and 8E8) could specifically react with recombinant F1K protein and FAdV-4 infected cells. Sequencing analysis of the variable regions revealed that the mAbs were from different hybridoma cell lines. Peptide array scanning of overlapping peptides covering the knob domain of fiber-1 protein identified three potential B-cell epitopes, with mAbs 5C10 and 8D8 recognizing the same epitope but possessing sequence differences in their complementarity-determining regions. Subsequently, truncations of the three identified peptides were performed to determine their precise locations of the epitopes, and alanine-scanning mutagenesis was conducted to define essential amino acids of the epitopes involved in binding by mAbs. Structural analysis indicated that two adjacent epitopes ^319^SDVGYLGLPPH^329^ and ^328^PHTRDNWYV^336^ were located on the surface of the knob domain, while epitope ^407^VTTGPIPFSYQ^417^ was situated in the center of the knob trimer. Taken together, the generation of mAbs and identification of linear B-cell epitopes on the knob domain of fiber-1 protein may contribute to the functional study of fiber-1 protein and the development of novel vaccines, therapeutics, and diagnostic methods for the control of hypervirulent FAdV-4 infection.

## 2 Materials and methods

### 2.1 *Cells*, *plasmid and virus*

Leghorn male hepatocellular (LMH) cells were cultured in Dulbecco’s Modified Eagle’s Medium/F12 (Gibco, NY, USA) with penicillin 100 U/ml and streptomycin 100 mg/ml (Solarbio, P1400). Myeloma cells (SP2/0) were cultured in Dulbecco’s Modified Eagle’s Medium (Solarbio, Beijing, China) supplemented with 10% heat-inactivated fetal bovine serum (FBS, Gibco, USA) at 37°C with 5% CO_2_. FAdV-4 ZZ strain (GenBank accession No. MN337322.1) was isolated from layers in our previous study and stored in the Key Laboratory of Animal Immunology of Henan Academy of Agricultural Sciences. The fiber-1 knob domain-containing plasmid pET-28a-F1K was synthesized by Shanghai Sangon Bioengineering Co., Ltd. (Shanghai, China).

### 2.2 Expression and purification of the fiber-1 knob protein

The plasmid PET-28a-F1K was transformed into the Chaperone Competent Cells pTf16/BL21 prepared using the Competent Cell Preparation Kit (TAKARA, Code No. 9139). Then, the *E. coli* cells were grown in Luria-Bertani (LB) medium at 37°C before induction of protein expression. L-arabinose was added to induce the expression of chaperone to prevent protein aggregation. The expression of F1K protein was induced by adding 1.0 mM isopropyl-beta-d-thiogalactopyranoside (IPTG) at 25°C for 12 h. After harvesting by centrifugation at 6,000 rpm for 20 min, the induced cells were resuspended using binding buffer (20 mM Tris-HCl, 150 mM NaCl, pH 7.5) and subjected to ultrasonication on ice. The supernatant was collected by centrifugation at 10,000 rpm for 10 min, filtered through a 0.22 um filter (Millipore, USA), and loaded onto a Ni-NTA agarose column for purification using the GE AKTA Purified Purification system (GE, USA). The unwanted proteins were washed from the column by washing buffer (20 mM Tris-HCl, 150 mM NaCl, 50 mM imidazole, pH 7.5), and the F1K protein was eluted by elution buffer (20 mM Tris-HCl, 150 mM NaCl, 100 mM imidazole, pH 7.5). The expression and purity of F1K protein were determined by 12.5% sodium dodecyl-sulfate polyacrylamide gel electrophoresis (SDS-PAGE). The bioactivity of F1K protein was analyzed by Western blot and dot-blot. After removing imidazole by dialysis against 10 mM Tris-HCl (pH 7.5), the recombinant F1K protein was concentrated by ultrafiltration, and the concentration was measured using a BCA protein quantification kit (CW biotech, Beijing, China).

### 2.3 Preparation of monoclonal antibodies against FAdV-4

Each of the three six-week-old female BALB/c mice was subcutaneously immunized with 20 μg of purified F1K protein formulated with Freund’s complete adjuvant (Sigma-Aldrich, Shanghai, China) and boosted twice with the same amount of F1K protein emulsified with Freund’s incomplete adjuvant at 2-week intervals. Serum antibody titers were determined by indirect enzyme-linked immunosorbent assay (ELISA). The mouse giving the highest antibody titer was injected intraperitoneally with 40 μg F1K protein three days before cell fusion. Then, the mouse was sacrificed and the splenocytes were harvested and fused with SP2/0 myeloma cells under the treatment of polyethylene glycol (PEG 1500, Roche). After cell fusion, positive hybridomas were screened by F1K protein-based indirect ELISA. Ascites in mice were produced for the best hybridomas and IgG was purified from the ascitic fluids using a Pierce™ protein A column (Thermo Fisher Scientific, USA). The subtypes of monoclonal antibodies were determined using a commercial isotyping kit (Proteintech, Wuhan, China).

### 2.4 Indirect ELISA

Briefly, 100 μL of the F1K protein (1.0 μg/ml) was coated onto the wells of ELISA plates (Nunc, Glostrup, Denmark) using 0.05 M carbonate–bicarbonate buffer (CBS, pH 9.6) at 4°C overnight. Then, the plates were washed three times with phosphate-buffered saline containing 0.05% Tween 20 (PBST) and blocked with 5% skimmed milk at 37°C for 2 h. The supernatant of the hybridoma cells was used as the primary antibody to incubate with the coated plates at 37°C for 1 h. After washing with PBST six times, horseradish peroxidase (HRP)-conjugated goat anti-mouse IgG (H+L) (Abbkine, AmyJet Scientific, Wuhan, China) was used as secondary antibodies to incubate with the plates at 37°C for 1 h. After washing, 3,3’,5,5’-tetramethylbenzidine (TMB) substrate (Amresco, Cleveland, OH, USA) was added for color development in the dark at room temperature for 10 min. Finally, the reaction was stopped with the addition of 2 M H_2_SO_4_, and the absorbance was read at a wavelength of 450 nm using a microplate reader (Omega, Germany).

### 2.5 Characterization of monoclonal antibodies

The reactivity of the monoclonal antibodies with F1K protein and FAdV-4 was confirmed using Western blot, dot-blot, and IFA. In Western blot and dot-blot, the protein or cell lysis from FAdV-4 infected cells was transferred onto polyvinylidene fluoride (PVDF) membranes or spotted on nitrocellulose (NC) membranes, respectively. Then, the membranes were blocked with 5% skimmed milk at 37°C for 1 h. Supernatants from the hybridoma cells or positive chicken serum against FAdV-4 were used as primary antibodies. HRP-conjugated goat anti-mouse IgG or HRP-conjugated goat anti-chicken IgY were used as secondary antibodies. Finally, the membranes were detected with NcmECL Ultra ECL (Enhanced Chemiluminescent, NCM BIO.CTD, China) reagent and visualized using a digital imaging system.

### 2.6 IFA

The LMH cells were seeded in 96-well cell culture plates and grown in an incubator at 37°C with 5% CO_2_. Then, the cells were infected with 0.001 multiplicity of infection (MOI) of FAdV-4 for 24 h. The plates were washed with phosphate-buffered saline (PBS) and fixed with cold methanol containing 3% H_2_O_2_ at -40°C for 30 min. After being sealed with 5% skimmed milk, the cells were incubated with the supernatants of the hybridoma cells at 37°C for 1 h. After washing with PBST, goat anti-mouse IgG (H + L)-Alexa Fluor 488 (1:1000 dilution) was added to incubate with the cells at room temperature for 1 h. After washing the cells with PBST for five times, 4’,6-diamidino-2-phenylindole (DAPI) solution (Solarbio, Beijing, China) was added to incubate with the cells as nucleus chromogen. After staining at room temperature for 10 min, the cells were observed and photographed under fluorescence microscopy (ZEISS, Germany).

### 2.7 Sequencing analysis of the variable regions of the mAbs

Total RNA of hybridoma cells grown in 24-well plates was extracted using RNAiso Plus reagent (Takara, Code No. 9109), and the cDNA was synthesized using PrimeScript™ RT Master Mix (Takara, Code No. RR036A). The variable domains of the heavy- and light-chain of the mAbs were amplified by polymerase chain reaction (PCR) (Jiang et al., 2021). The PCR products were ligated with pMD 18-T Vector using pMD™ 18-T Vector Cloning Kit (Takara, Code No. 6011) and sequenced by Shanghai Sangon Bioengineering Co., Ltd. (Shanghai, China). The sequences of the heavy- and light-chain variable regions were analyzed using IgBlast and IMGT/V-QUEST online software, and the complementarity-determining regions (CDRs) were marked after being aligned by the ClustalW method using DNAstar software.

### 2.8 Precise identification of linear B cell epitopes on F1K protein

To identify potential B cell epitopes on the knob domain (221-431 aa) of fiber-1 protein, 26 overlapping peptides with 7 amino acid offsets were synthesized by Gill Bio (Shanghai, China). A cysteine (C) was added to the synthesized peptide at the amino-terminus for conjugation with bovine serum albumin (BSA). The peptide was completely freeze-dried and existed as a white, powdery solid. The purity of all synthesized peptides was over 95%. Before use, the peptide was dissolved in sterile water to a final concentration of 5 mg/ml. Peptide-based indirect ELISA and dot-blot were used to identify the reaction between the peptides and mAbs. In peptide ELISA, synthetic peptides were coated onto 96-well ELISA plates (250 ng/well) and tested according to the procedures described in indirect ELISA. In dot-blot, peptides were diluted to 1 mg/ml, spotted onto nitrocellulose (NC) membranes (1μL), and tested as described above.

To determine the crucial amino acids of the identified epitope peptides, alanine-scanning mutagenesis was performed in which key amino acids of peptides 13-1, 14-2, and 24-1 were substituted one by one with alanine (A). Subsequently, the reactivity of the mutant peptides was tested by dot-blot with the original peptides as positive controls. For identification, the mAbs 5C10, 6F8, 8D8, and 8E8 were used as primary antibodies to analyze their corresponding peptides.

### 2.9 Analysis of the spatial structure of the epitopes

The tertiary structure of the knob trimer of FAdV-4 fiber-1 protein was obtained from the PDB database (PDB: ID 7X5T). The spatial structural property of the identified linear epitope was analyzed using the PyMOL software (Version 2.5.2, Schrödinger, LLC.).

## 3 Results

### 3.1 Preparation of recombinant F1K *protein*

**Figure 1:**
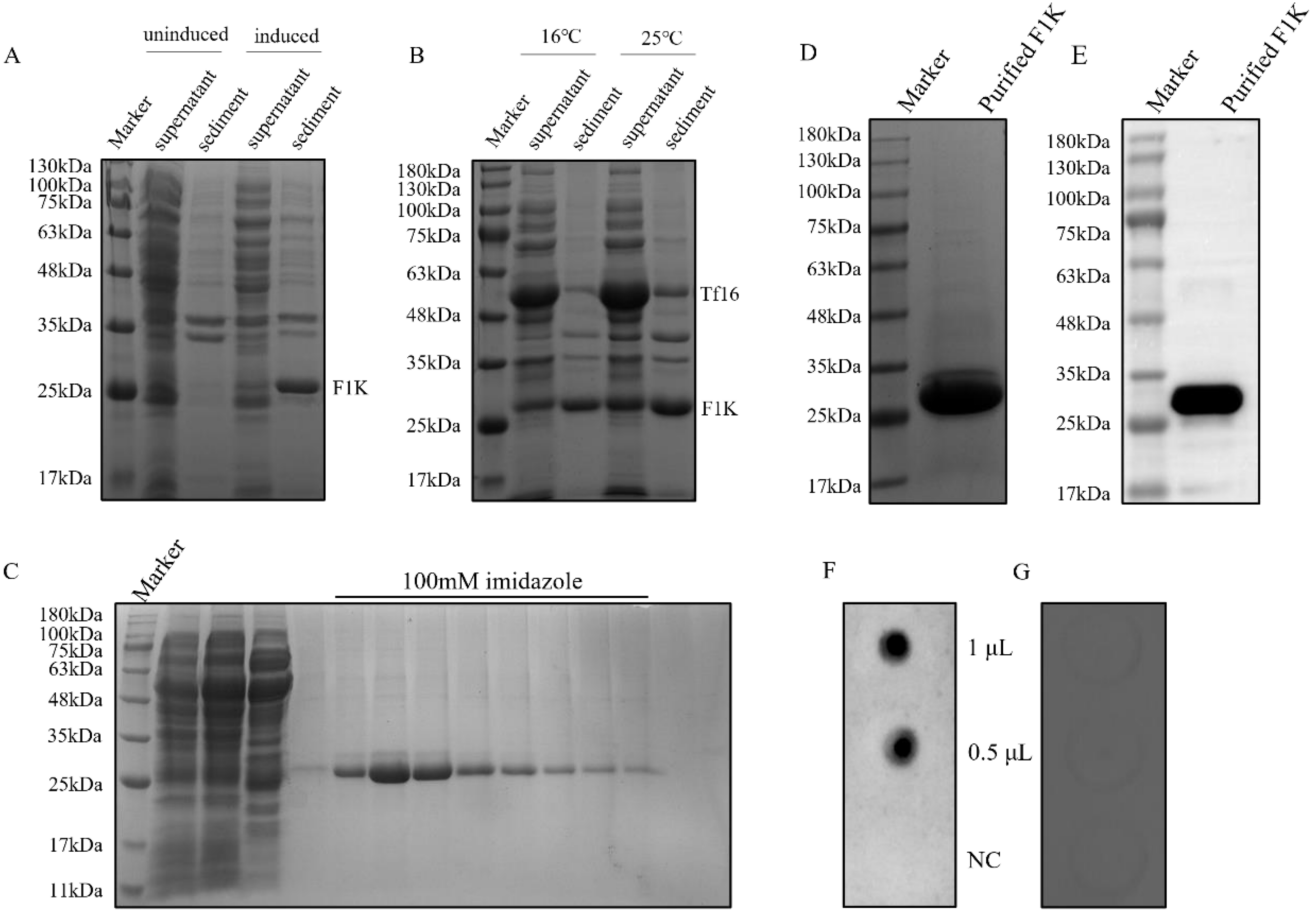
Preparation and identification of recombinant F1K protein. **(A)** *E. coli* cells harboring pET-28a-F1K were cultured in LB media and induced with IPTG. Expression of F1K protein was detected in the supernatant and sediment of cells after ultrasonication. Uninduced cells were used as controls. **(B)** *E. coli* cells harbouring pET-28a-F1K were cultured in LB media containing L-arabinose and induced with IPTG at 16°C and 25°C, respectively. Expression of F1K protein was detected in the supernatant and sediment of cells after ultrasonication. **(C)** Purification of F1K protein using Ni-NTA affinity chromatography. F1K protein was eluted from the Ni-NTA agarose column with an elution buffer containing 100 mM imidazole. **(D)** The eluted F1K protein was concentrated by ultrafiltration and its purity was examined by SDS-PAGE. **(E)** The reaction between F1K protein and anti-His-tag monoclonal antibody in Western blot. **(F, G)** The reaction between F1K protein and positive chicken serum against FAdV-4 and normal chicken serum; NC meant negative control which represented the reaction between the serum and BSA.

Expression of recombinant F1K protein in *E. coli* cells was induced with IPTG and detected in cell supernatant and sediment after ultrasonication, respectively. As shown in **Figure 1A**, recombinant F1K protein (26 kDa) was prone to aggregation and formed inclusion bodies when *E. coli* cells were induced only with IPTG. When the cells were cultured in LB media containing L-arabinose and induced with IPTG, the expression of both chaperone and F1K protein was obtained. And a large amount of water-soluble F1K protein was harvested in the presence of the molecular chaperone, Tf16 (**Figure 1B**). Meanwhile, expression of recombinant F1K protein was induced at 16°C and 25°C and it showed that more F1K protein appeared in the supernatant when the cells cultured in L-arabinose-containing media were induced with 1.0 mM IPTG at 25°C (**Figure 1B**). Ni-NTA affinity chromatography was used to purify F1K protein based on the interaction between the poly-histidine tag of recombinant F1K protein and the affinity resin. Buffers containing 20 mM imidazole were used to remove unwanted proteins from the Ni-NTA agarose column. Recombinant F1K protein was eluted with an elution buffer containing 100 mM imidazole (**Figure 1C**). After ultrafiltration, the purity of recombinant F1K protein was examined by SDS-PAGE and there was a clear band of purified F1K protein (**Figure 1D**). The reactivity of recombinant F1K protein was analyzed by Western blot and dot-blot. Western blot showed that recombinant F1K protein could react with anti-His-tag monoclonal antibody (**Figure 1E**). Dot-blot showed that recombinant F1K protein could be recognized by positive chicken serum against FAdV-4 and did not react with normal chicken serum (**Figures 1F, G**).

### 3.2 Generation and characterization of monoclonal antibodies

**Figure 2:**
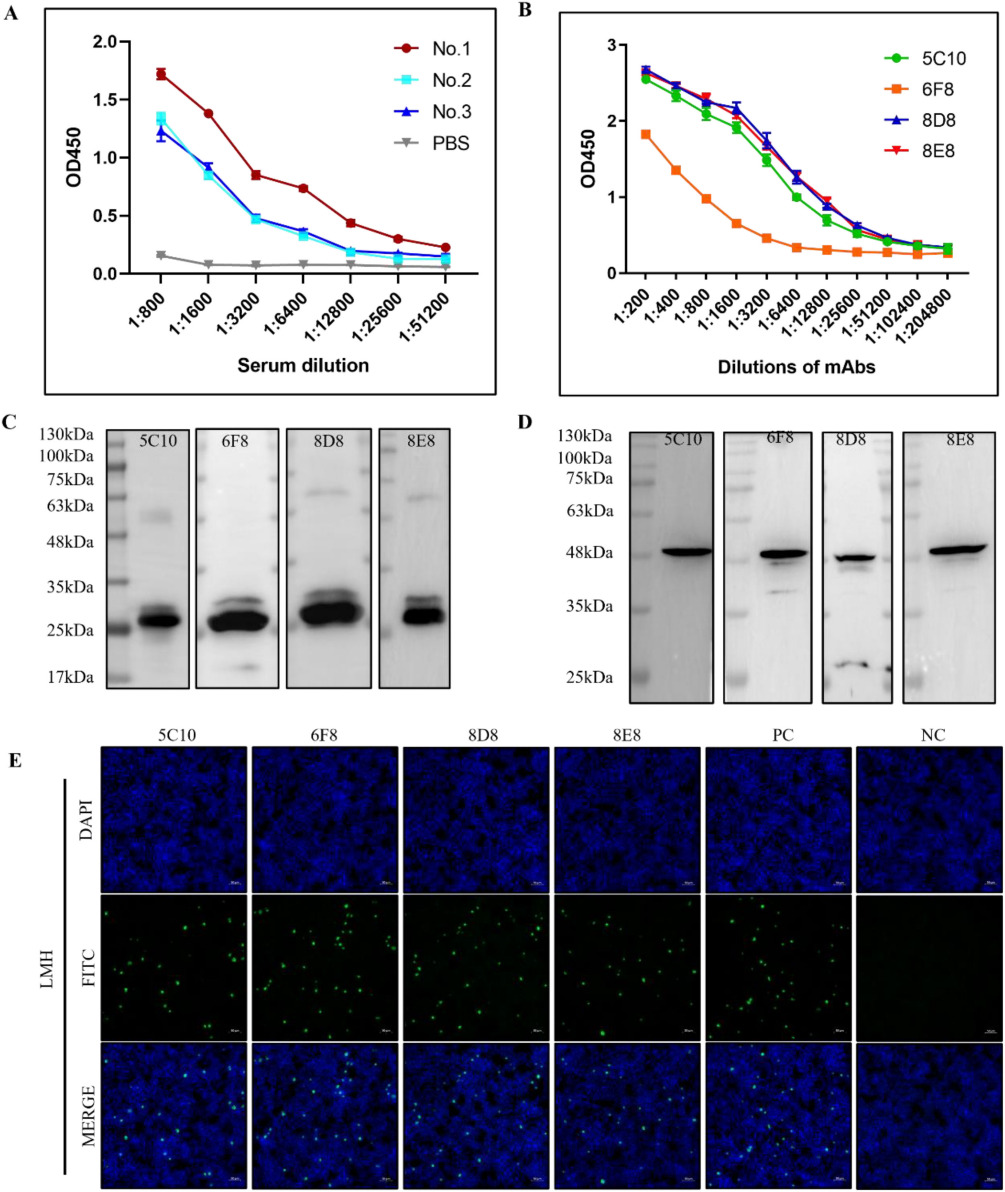
Characterizations of mAbs against FAdV-4. **(A)** Determination of antibody titers. Mice were immunized three times with F1K protein or PBS and antibody titers of serum samples were detected by indirect ELISA. **(B)** Titers of the mAbs in ELISA. **(C, D)** The reactivity of mAbs with the recombinant F1K protein and the fiber-1 protein from the lysis of FAdV-4 infected LMH cells, respectively. **(E)** The reactivity of mAbs 5C10, 6F8, 8D8, and 8E8 with LMH cells infected with FAdV-4 ZZ strain at an MOI of 0.001. PC represented serum from immunized mouse; NC represented serum from normal mouse. Scale bars, 50 μm.

After three immunizations, serum antibody titers were determined in immunized mice (**Figure 2A**). The mouse with the highest antibody titer was selected for preparation of monoclonal antibodies. After screening with F1K protein-based indirect ELISA, four hybridoma cell lines that can specifically recognize F1K protein were selected and named 5C10, 6F8, 8D8, and 8E8, respectively. The titers of the mAbs in ELISA were determined to be 1:12800, 1:3200, 1:12800, and 1:12800, respectively (**Figure 2B**). In addition, Western blot showed that all the mAbs could react with both recombinant F1K protein and fiber-1 protein extracted from the cell lysis of FAdV-4 infected LMH cells (**Figures 2C, D**). The mAbs were further characterized by testing their reactivity with FAdV-4 infected cells using IFA, and it was found that all the mAbs could react obviously with FAdV-4 in LMH cells (**Figure 2E**). Taken together, these results suggested that all the mAbs recognized linear epitopes on recombinant F1K protein and native fiber-1 protein. The heavy chain subtypes of 5C10, 8D8, and 8E8 were determined to be IgG2b, and that of 6F8 belonged to IgG1. The light chain subtype of all mAbs was kappa (**Table 1**).

**Table 1:** Characterization of monoclonal antibodies against FAdV-4.

	Monoclonal antibodies			
	5C10	6F8	8D8	8E8
Subclasses of Ig	IgG2b	IgG1	IgG2b	IgG2b
Types of light chains	κ			
Types of epitopes	Linear			
Ascitic fluid titers	>10^6^			

### 3.3 Sequence analysis of FAdV-4 specific mAbs

**Figure 3:**
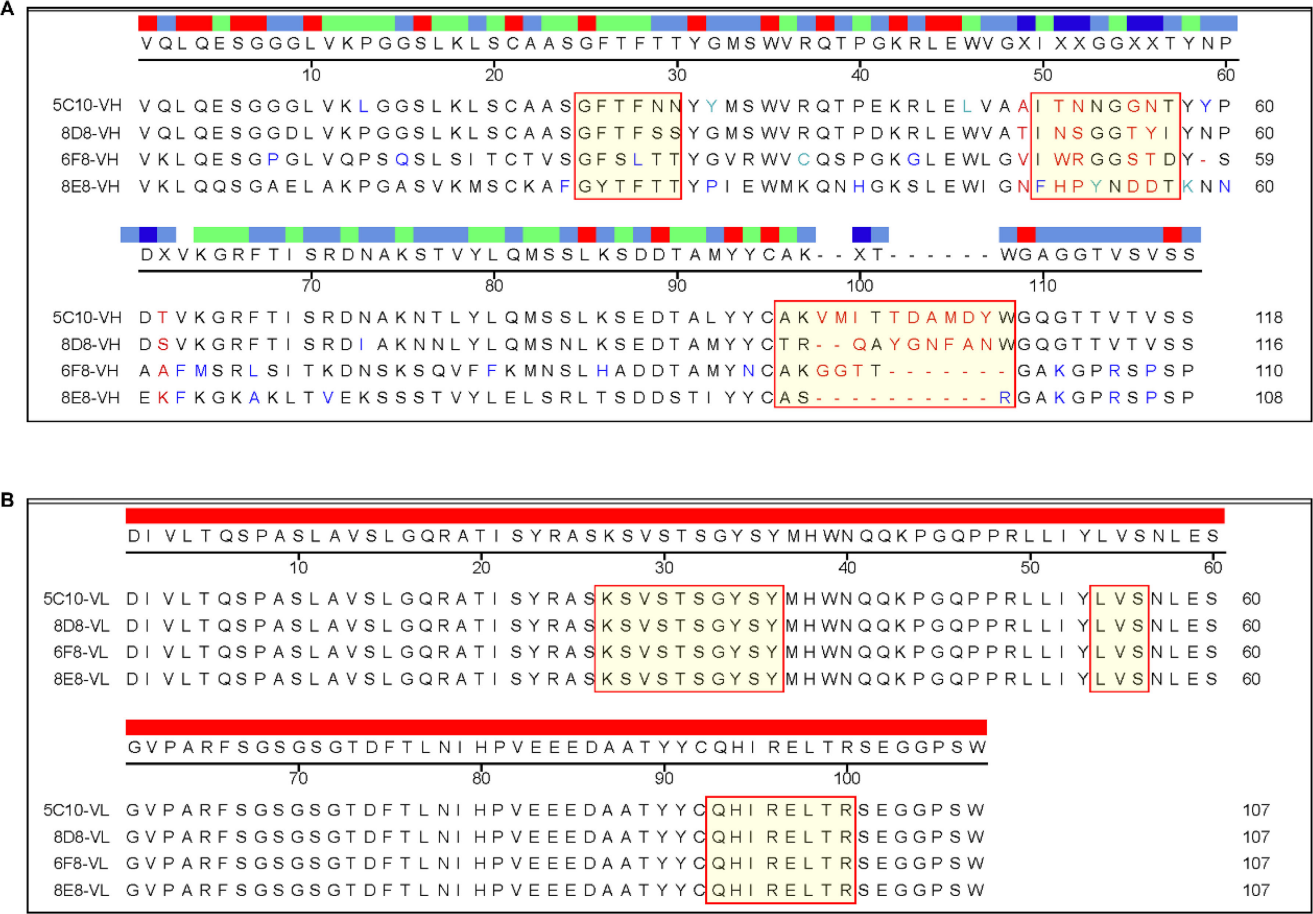
Sequencing analysis of the variable regions of the mAbs. **(A)** Sequence alignment of the variable region of heavy chain. The complementarity-determining regions (CDRs) were labeled with special red boxes. **(B)** Sequence alignment of the variable region of light chain. Multiple alignment was performed using DNAstar software.

The variable regions of all mAbs were amplified and sequenced. As shown in **Figure 3**, the mAbs had different VH sequences from each other. However, the VL sequences were identical in all mAbs (**Figure 3**). The VH sequences of 5C10 and 8D8 were similar, with some differences in the CDRs, especially the CDR-H3 region.

### 3.4 Identification of the B cell epitopes on F1K protein

**Figure 4:**
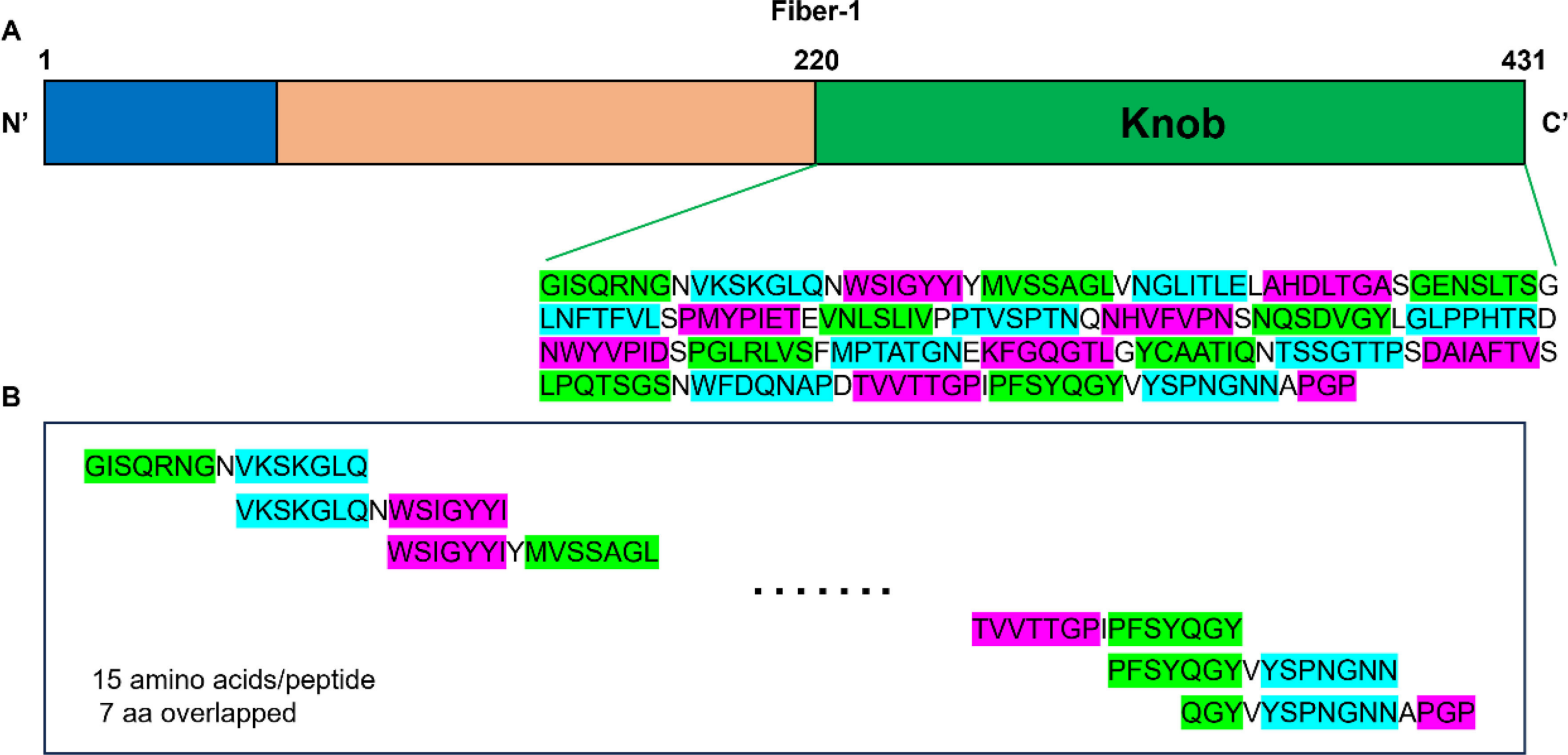
Design of a peptide array based on the sequence of the knob domain of fiber-1 protein. **(A)** Schematic diagram of the structure of the fiber-1 protein. The location and amino acid sequence of the knob domain were labeled. **(B)** The knob domain of fiber-1 protein was divided into 26 overlapping peptides. Each peptide had 15 amino acids with 7 amino acids overlap. The last peptide had 11 amino acids overlap with its previous one.

**Figure 5:**
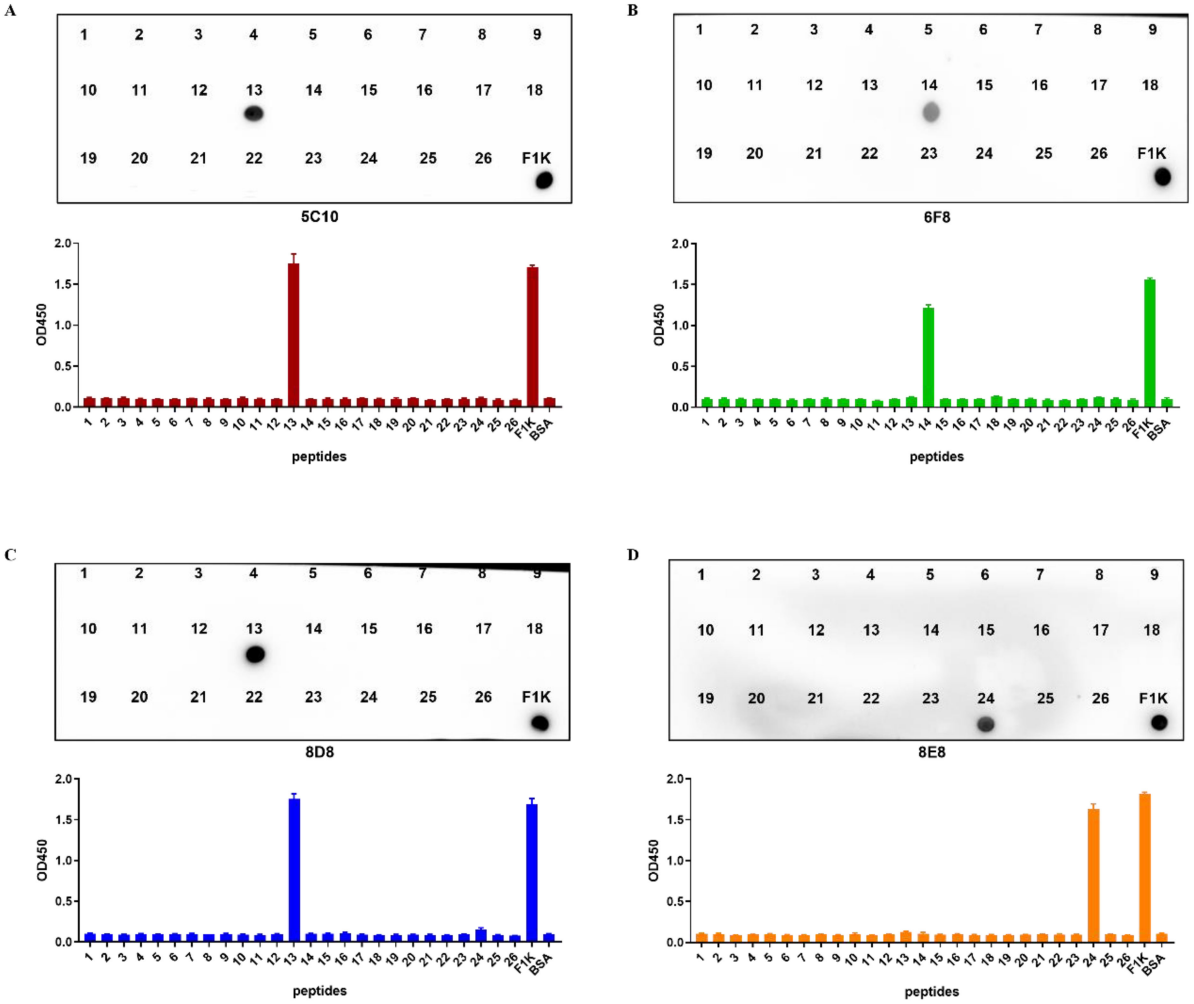
Identification of B cell epitopes recognized by the mAbs. **(A–D)** Indirect ELISA and dot-blot were used to identify the reactivity of four mAbs with the 26 overlapping peptides, respectively. The data were collected from three independent replicates, and shown as means with error bars. Recombinant F1K protein was used as positive control. BSA was used as blank control.

As shown in **Figure 4** and **Table 2**, 26 overlapping peptides with 7 amino acid offsets were designed and synthesized based on the amino acid sequence of the fiber-1 knob domain. The locations of epitopes were mapped by testing the reactions of all the four mAbs with the overlapping peptides in peptide-based indirect ELISA. It was found that mAbs 5C10 and 8D8 reacted specifically with peptide No. 13 (^317^NQSDVGYLGLPPHTR^331^). The mAb 6F8 reacted specifically with peptide No. 14 (^325^GLPPHTRDNWYVPID^339^) and mAb 8E8 specifically targeted peptide No. 24 (^405^TVVTTGPIPFSYQGY^419^). The OD_450_ values of the reactive peptides with mAbs were similar to those of recombinant F1K protein and mAbs. In addition, dot-blot showed consistent results with peptide ELISA (**Figures 5A–D**).

**Table 2:** Overlapping peptides covering the knob domain of FAdV 4 fiber-1 protein.

No.	Peptide sequences	No.	Peptide sequences	No.	Peptide sequences
1	GISQRNGNVKSKGLQ	10	VNLSLIVPPTVSPTN	19	YCAATIQNTSSGTTP
2	VKSKGLQNWSIGYYI	11	PTVSPTNQNHVFVPN	20	TSSGTTPSDAIAFTV
3	WSIGYYIYMVSSAGL	12	NHVFVPNSNQSDVGY	21	DAIAFTVSLPQTSGS
4	MVSSAGLVNGLITLE	13	NQSDVGYLGLPPHTR	22	LPQTSGSNWFDQNAP
5	NGLITLELAHDLTGA	14	GLPPHTRDNWYVPID	23	WFDQNAPDTVVTTGP
6	AHDLTGASGENSLTS	15	NWYVPIDSPGLRLVS	24	TVVTTGPIPFSYQGY
7	GENSLTSGLNFTFVL	16	PGLRLVSFMPTATGN	25	PFSYQGYVYSPNGNN
8	LNFTFVLSPMYPIET	17	MPTATGNEKFGQGTL	26	QGYVYSPNGNNAPGP
9	PMYPIETEVNLSLIV	18	KFGQGTLGYCAATIQ		

**Figure 6:**
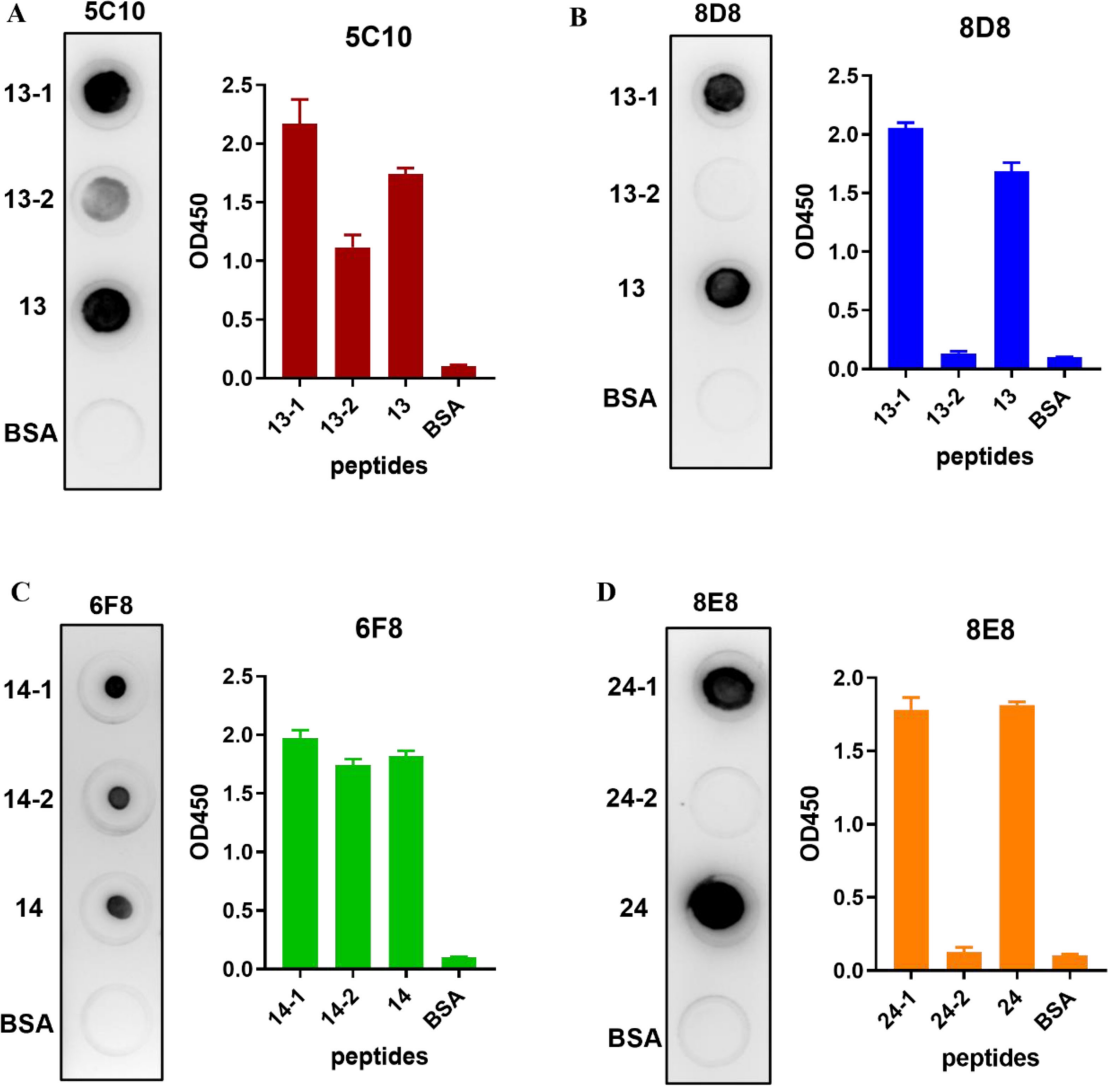
Identification of truncated peptides recognized by the mAbs. **(A–D)** The reactions of four mAbs with the truncated peptides were identified using dot-blot and indirect ELISA, respectively. The ELISA data were collected from three independent replicates and shown as means with error bars. The untruncated peptide was used as positive control. BSA was used as blank control.

Subsequently, truncations of the three identified peptides were prepared to determine the precise locations of epitopes (**Table 3**). Reactions of the four mAbs with the truncated peptides were identified using dot-blot and peptide-based indirect ELISA (**Figures 6A-D**). It was shown that mAb 5C10 and 8D8 could only react with peptide No. 13-1. The mAb 6F8 could react with both peptide No. 14-1 and peptide No. 14-2. The mAb 8E8 specifically recognized peptide No. 24-1 but did not react with peptide No. 24-2.

**Table 3:** Amino acid sequence of the truncated peptides (No. 13, 14, and 24).

Peptide name	Peptide sequence
13-1 13-2 14-1 14-2 24-1 24-2	CSDVGYLGLPPH CDVGYLGLPP CPPHTRDNWYVP CPHTRDNWYV CVTTGPIPFSYQ CTTGPIPFSY

### 3.5 Identification of the core binding sites of the identified epitopes

**Figure 7:**
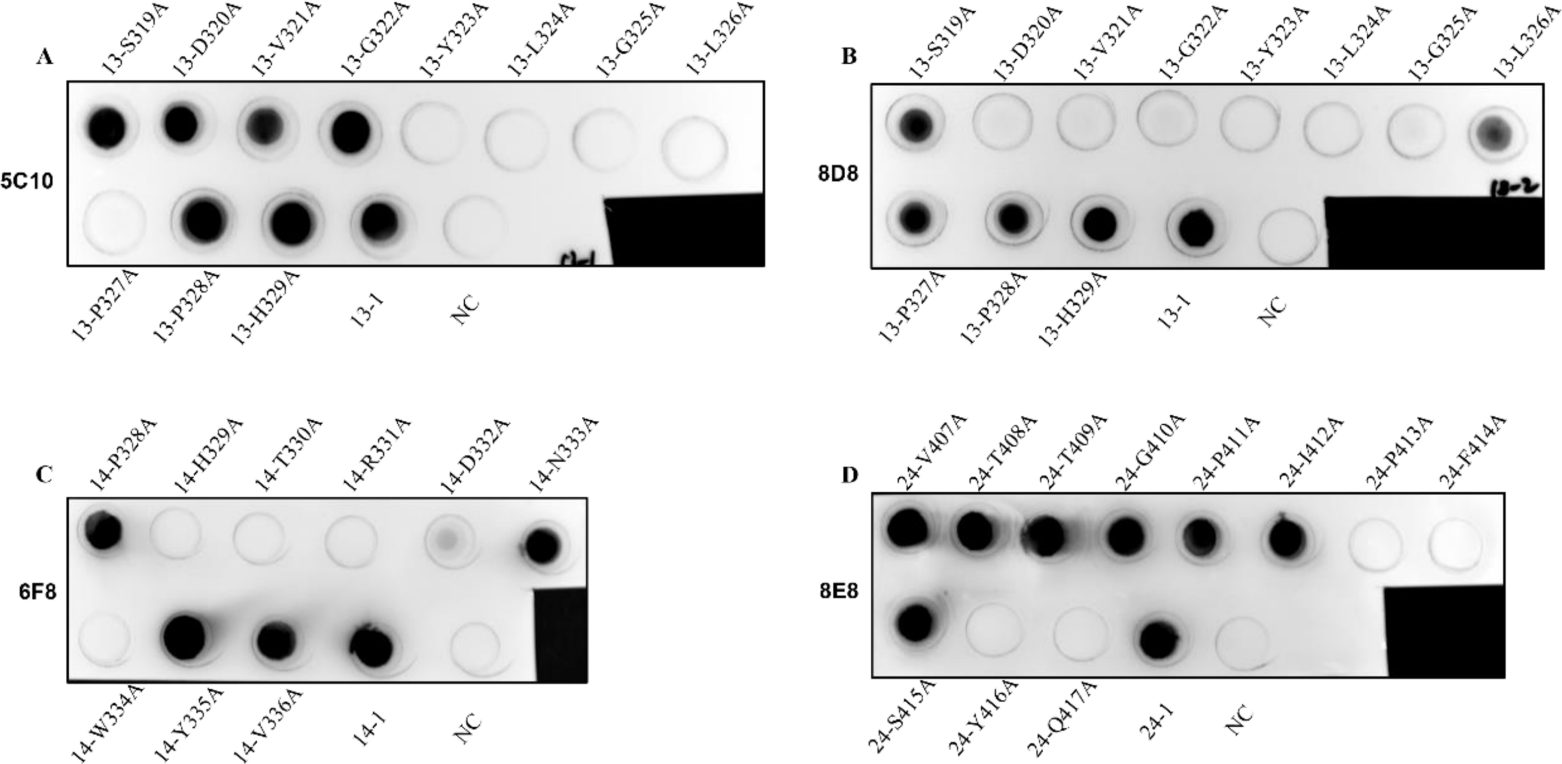
Identification of the key amino acid residues of the epitopes for binding to mAbs. **(A–D)** The binding ability of the mutant peptides with the corresponding mAbs 5C10, 8D8, 6F8, and 8E8 was determined by dot-blot, respectively. The unmutated peptides were used as positive control. BSA was used as negative control (NC).

Alanine-scanning mutagenesis was performed to define the crucial amino acid residues of the epitopes involved in binding by mAbs 5C10, 6F8, 8D8, and 8E8. Each residue of the truncated peptides (No. 13-1, 14-2, and 24-1) was sequentially substituted by alanine (**Table 4**). The reactivity of these mutant peptides with the corresponding mAbs was determined by dot-blot (**Figure 7**). As shown in **Figures 7A** and **B**, the key amino acid of epitope No. 13-1 (^319^SDVGYLGLPPH^329^) recognized by mAbs 5C10 and 8D8 was different. There was no binding of epitope No. 13-1 with mAb 5C10 when the ^323^Y, ^324^L, ^325^G, ^326^L, and ^327^P were substituted to the alanine, respectively (**Figure 7A**). The substitution of residues ^320^D, ^321^V, ^322^G, ^323^Y, ^324^L, and ^325^G with alanine resulted in the loss of reactivity between peptide No. 13-1 and mAb 8D8 (**Figure 7B**). Meanwhile, it was shown that residues ^329^H, ^330^T, ^331^R, ^332^D, ^333^N, and ^334^W were the critical amino acids for the binding between peptide No. 14-2 and mAb 6F8 (**Figure 7C**). Amino acid residues ^413^P, ^414^F, ^416^Y, and ^417^Q were identified as essential for the binding between epitope No. 24-1 and mAb 8E8 (**Figure 7D**).

**Table 4:** Sequences of mutant peptides generated by alanine-scanning mutagenesis.

Name	Peptide sequences	Name	Peptide sequences	Name	Peptide sequences
13-S319A 13-D320A 13-V321A 13-G322A 13-Y323A 13-L324A 13-G325A 13-L326A 13-P327A 13-P328A 13-H329A	CADVGYLGLPPH CSAVGYLGLPPH CSDAGYLGLPPH CSDVAYLGLPPH CSDVGALGLPPH CSDVGYAGLPPH CSDVGYLALPPH CSDVGYLGAPPH CSDVGYLGLAPH CSDVGYLGLPAH CSDVGYLGLPPA	14-P328A 14-H329A 14-T330A 14-R331A 14-D332A 14-N333A 14-W334A 14-Y335A 14-V336A	CAHTRDNWYV CPATRDNWYV CPHARDNWYV CPHTADNWYV CPHTRANWYV CPHTRDAWYV CPHTRDNAYV CPHTRDNWAV CPHTRDNWYA	24-V407A 24-T408A 24-T409A 24-G410A 24-P411A 24-I412A 24-P413A 24-F414A 24-S415A 24-Y416A 24-Q417A	CATTGPIPFSYQ CVATGPIPFSYQ CVTAGPIPFSYQ CVTTAPIPFSYQ CVTTGAIPFSYQ CVTTGPAPFSYQ CVTTGPIAFSYQ CVTTGPIPASYQ CVTTGPIPFAYQ CVTTGPIPFSAQ CVTTGPIPFSYA

### 3.6 Structural analysis of the epitopes

**Figure 8:**
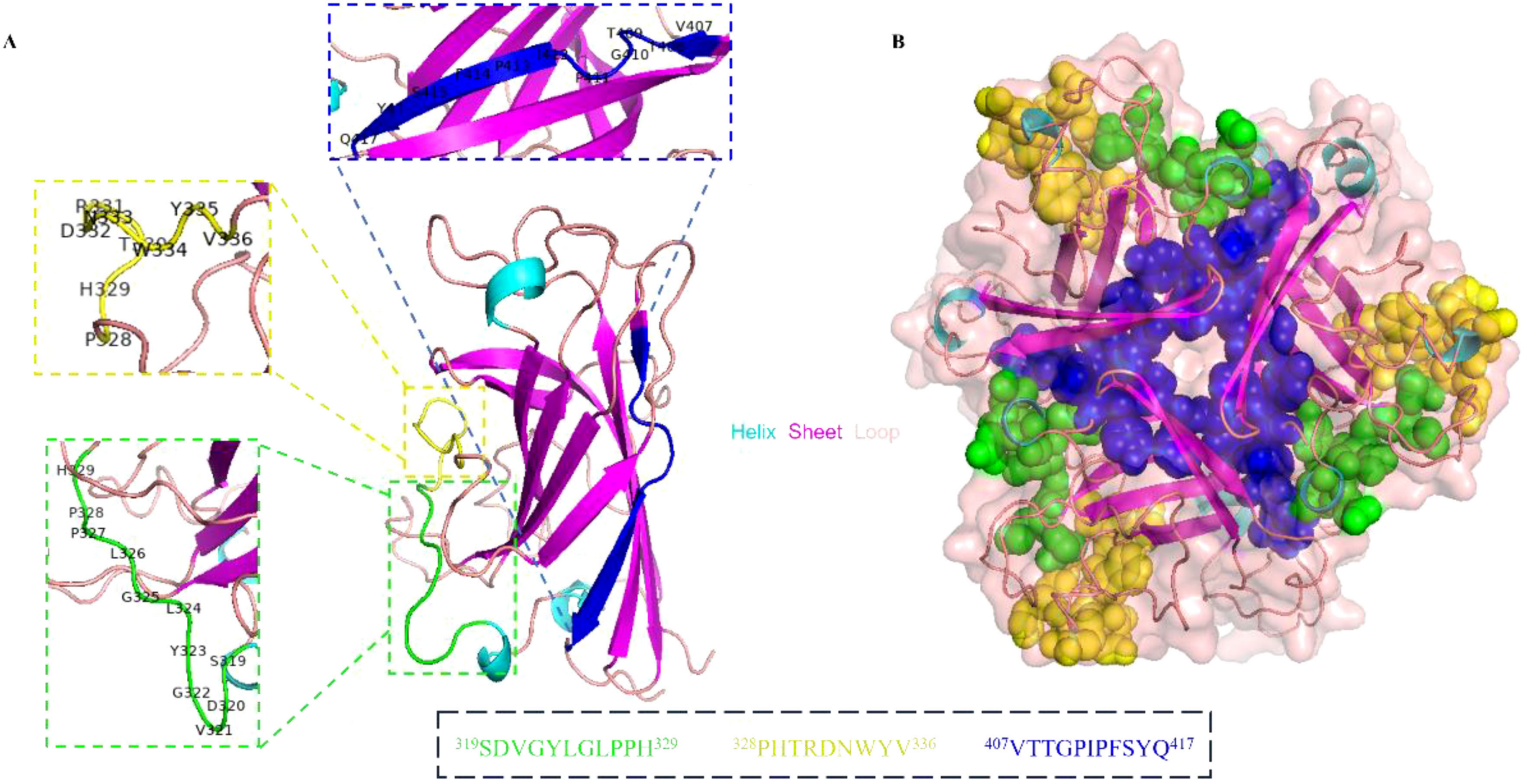
Spatial structure analysis of the epitopes. **(A)** The cartoon representation of the precise linear epitopes on the knob monomer. The epitopes were displayed in different colors, epitope No. 13-1 (^319^SDVGYLGLPPH^329^) in green, epitope No. 14-2 (^328^PHTRDNWYV^336^) in yellow, and epitope No. 24-1 (^407^VTTGPIPFSYQ^417^) in blue. **(B)** The spatial structure of epitopes was displayed on knob trimer in a sphere form.

The spatial distribution of the identified linear epitopes was analyzed using the PyMOL software. As shown in **Figure 8A**, two continuous epitopes No. 13-1 (^319^SDVGYLGLPPH^329^) and No. 14-2 (^328^PHTRDNWYV^336^) existed as the protein loop of the knob domain. Epitope No. 24-1 (^407^VTTGPIPFSYQ^417^) was located near the C-terminus of the knob domain, and existed as the β sheet in the secondary structure. Epitopes No. 13-1 (^319^SDVGYLGLPPH^329^) and No. 14-2 (^328^PHTRDNWYV^336^) were located in different faces with No. 24-1 (^407^VTTGPIPFSYQ^417^) on the knob monomer. The cartoon representation further proved that the identified B cell epitopes were linear. Based on the trimeric structure of the knob domain, epitopes No. 13-1 (^319^SDVGYLGLPPH^329^) and No. 14-2 (^328^PHTRDNWYV^336^) were exposed on the surface and No. 24-1 (^407^VTTGPIPFSYQ^417^) was located in the center of the knob trimer (**Figure 8B**).

## 4 Discussion

Recently, the emergence of a novel genotype of FAdV-4 in China has brought significant economic losses to the poultry industry (Li et al., 2016; Niu et al., 2018). Until now, HHS continues to occur in broiler flocks and there are no effective commercial vaccines or drugs to prevent its transmission (Li et al., 2017; Niu et al., 2022). As a major surface structural protein of FAdVs, the fiber protein has been reported to carry immunodominant B cell epitopes that can effectively induce the production of neutralizing antibodies, suggesting its potential use as the main target for the development of vaccines (Lu et al., 2019; Schachner et al., 2022; Chen et al., 2023). Fiber-1 protein, the shorter one of the two fibers of FAdV-4, has been reported to interact with the cell receptor to mediate viral infection (Marek et al., 2012; Pan et al., 2020). In addition, it has been demonstrated that recombinant fiber-1 protein expressed in the baculovirus expression system not only induced high levels of neutralizing antibodies in chickens but also avoided the occurrence of clinical signs or histopathological changes after challenging with virulent FAdV-4 (Watanabe et al., 2023). Meanwhile, immunization with recombinant fiber-1 protein expressed in *E. coli* cells has also been reported to provide complete protection against the lethal challenge of FAdV-4 in chickens (Wang et al., 2018). To be noted, although a previous study showed that vaccination with recombinant fiber-1 protein prepared from the baculovirus expression system resulted in partial protection in chickens against FAdV-4 challenge and led to a mortality of 38%, it did not negate the significant role of fiber-1 protein in the development of subunit vaccines against FAdV-4 (Schachner et al., 2014).

The fiber protein of FAdV-C has been proven to be different from other species of FAdVs, which suggested its potential use as an important target for developing differential diagnostic serological assays (Marek et al., 2012). In a previous report, two mAbs generated by immunizing mice with recombinant fiber-1 protein of FAdV-4 were used in the development of a sandwich ELISA, which was shown to be able to selectively identify the presence of FAdV-C from other FAdVs (Shao et al., 2019). The knob domain of fiber protein is located on the external surface of the virion and may interact directly with cell receptors to facilitate FAdV-4 infection (Song et al., 2024). In this study, the knob domain of the fiber-1 protein (F1K) was expressed in *E. coli* cells. To avoid the formation of inclusion bodies, the molecular chaperone, Tf16, was used to facilitate the harvest of water-soluble F1K protein. Through co-expression of Tf16 with F1K protein and lowering the induction temperature from 37°C to 25°C, large amount of water-soluble F1K protein appeared in the supernatant after ultrasonication. Then, recombinant F1K protein was purified through Ni-NTA chromatography and used to immunize BALB/c mice. Subsequently, four mAbs (5C10, 6F8, 8D8, and 8E8) were generated and characterized. ELISA revealed that all the mAbs possessed high-affinity binding with recombinant F1K protein. Western blot showed that all mAbs could react specifically with both recombinant F1K protein and fiber-1 protein extracted from infected cells. The reactions between F1K protein and mAbs in ELISA and Western blot indicated that the epitope recognized by mAbs might be linear. In addition, IFA showed that all the mAbs were able to react with FAdV-4 infected LMH cells. These results indicated all mAbs were FAdV-4 specific and could be used for the identification of B-cell epitopes. In addition, the variable regions of these mAbs were sequenced and the results revealed that they were from different hybridoma cell lines.

Epitopes are the basic units of an antigen that bind to B-cell receptors and stimulate the production of antibodies. The identification and precise location of epitopes are fundamental to understanding the structure and function of antigens and the development of epitope-based diagnostic reagents and vaccines (Lang et al., 2016; Sanami et al., 2020). Previously, Pan et al. generated 10 mAbs against the hexon L1 of FAdV-4 and identified three linear epitopes including one specific epitope on FAdV-4 and two shared epitopes of FAdV-C species (Pan et al., 2018). In addition, another study also identified a linear epitope on the hexon protein of FAdV-4, which was proved to be conserved across all the FAdVs (Liu et al., 2021a). To date, no studies have been conducted to identify potential epitopes on the fiber-1 protein of FAdV-4, despite the importance of the protein in the development of diagnostic methods and vaccines. In this study, 26 overlapping synthetic peptides covering the knob domain of fiber-1 protein were designed and used to identify potential linear epitopes recognized by the generated mAbs. After primary screening using peptide-based ELISA and dot-blot, peptides No. 13, 14, and 24 were recognized by four mAbs. Among them, peptide No. 13 could be recognized by mAbs 5C10 and 8D8. This might be because these two mAbs had similar variable regions as shown in the sequencing analysis of the variable domains of the heavy- and light-chain. Subsequently, the three peptides were truncated to determine the precise location of the B-cell epitopes recognized by these mAbs. As shown in **Figure 6**, the truncated peptides No. 13-1 (^319^SDVGYLGLPPH^329^), No. 14-2 (^328^PHTRDNWYV^336^), and No. 24-1 (^407^VTTGPIPFSYQ^417^) were identified by mAbs 5C10 and 8D8, 6F8, as well as 8E8, respectively.

Subsequently, alanine-scanning mutagenesis was used to guide the design of the specific mutant peptide and identify the amino acids that play a crucial role in the interaction between the epitopes and the mAbs. It was demonstrated that the key amino acids of epitope No. 13-1 (^319^SDVGYLGLPPH^329^) involved in binding to mAbs 5C10 and 8D8 were different. Mutations of ^323^Y, ^324^L, ^325^G, ^326^L, and ^327^P to alanine resulted in the loss of binding between peptide No. 13-1 (^319^SDVGYLGLPPH^329^) and mAb 5C10. In contrast, amino acid residues ^320^D, ^321^V, ^322^G, ^323^Y, ^324^L, and ^325^G were confirmed as the key amino acids for the binding between peptide No. 13-1 (^319^SDVGYLGLPPH^329^) and mAb 8D8. The reactivity of epitope No. 14-2 (^328^PHTRDNWYV^336^) with mAb 6F8 disappeared when the amino acid residues of ^329^H, ^330^T, ^331^R, ^332^D, and ^334^W were mutated into alanine, respectively. Meanwhile, amino acids ^413^P, ^414^F, ^416^Y, and ^417^Q were identified as the key residues of epitope No. 24-1 (^407^VTTGPIPFSYQ^417^) for binding to mAb 8E8.

In addition, the spatial structure of identified linear epitopes was visualized and analyzed using PyMOL software. It was shown that epitopes ^319^SDVGYLGLPPH^329^ and ^328^PHTRDNWYV^336^ were located in adjacent regions and existed as the protein loop on the secondary structure of the knob domain. Meanwhile, both two epitopes were exposed on the surface of the fiber-1 knob trimer. These results suggested that the region containing epitopes ^319^SDVGYLGLPPH^329^ and ^328^PHTRDNWYV^336^ might be the immunodominant region of the knob domain. For epitope ^407^VTTGPIPFSYQ^417^, it was exposed on the opposite side of the other two epitopes. Further, it was located close to the C-terminus of the knob domain and inside the knob trimer.

## 5 Conclusions

In summary, four mAbs against FAdV-4 were generated using hybridoma technology. Sequencing analysis of the variable regions showed all the mAbs were distinct from each other. Furthermore, three novel B-cell linear epitopes (^319^SDVGYLGLPPH^329^, ^328^PHTRDNWYV^336^, and ^407^VTTGPIPFSYQ^417^) in the knob domain of fiber-1 protein were defined using the mAbs and the key amino acid residues in these linear epitopes involved in binding by mAbs were identified using alanine-scanning mutagenesis. The spatial structure of these epitopes was demonstrated based on the structure of the fiber-1 knob protein (PDB: ID 7X5T). This was the first study to identify B-cell epitopes on the knob domain of FAdV-4 fiber-1 protein. The generated mAbs and identification of novel B-cell epitopes could greatly promote the development of subunit vaccines, therapeutics, and diagnostic methods for the control of FAdVs.

## Data Availability

The datasets presented in this study can be found in online repositories. The names of the repository/repositories and accession number(s) can be found in the article/supplementary material.
